# Effects of 4 mg and 8 mg Dexamethasone Added to Intrathecal Bupivacaine on Perioperative Analgesia Among Adult Orthopedic Patients at Sodo Christian Hospital: A Prospective Cohort Study

**DOI:** 10.1155/2024/8872988

**Published:** 2024-10-30

**Authors:** Amanuel Essayas, Mebratu Legesse, Mebratu Tila, Ashagire Sintayhu, Eyosiyas Abreham, Getahun Dendir

**Affiliations:** ^1^School of Anesthesia, Wolaita Soddo University, School of Anesthesia, Wolaita, Soddo, Ethiopia; ^2^Department of Pharmacy, Wolaita Soddo University, Wolaita, Soddo, Ethiopia; ^3^Internal Medicine, School of Medicine, College of Health Science and Medicine, Wolaita Sodo University, Wolaita, Sodo, Ethiopia

**Keywords:** dexamethasone, intrathecal bupivacaine, orthopedic pts, perioperative analgesia

## Abstract

**Background:** Several adjuvant drugs have been tried to prolong spinal anesthesia block. Currently, dexamethasone appears to be effective in extending the duration of sensory block and enhancing analgesia during surgery. It is unclear, however, whether administering dexamethasone at a dose of 8 mg offers any advantages over administering it at a dose of 4 mg.

**Objective:** To compare the effect of adding 4 and 8 mg dexamethasone to intrathecal bupivacaine on perioperative analgesia among adult orthopedic patients at Sodo Christian Hospital from June 1 to October 31, 2021.

**Methodology:** A total of 178 adult patients undergoing elective orthopedic surgery were randomly assigned to one of the two groups through a prospective cohort research design. A systematic random sampling method was used. For analysis, data were imported into EpiData v.4.6 and exported to SPSS v.25. Levene's test was used to verify homogeneity of variance, whereas the Shapiro–Wilk test was used to assess data distribution. The Mann–Whitney test and the independent sample *t*-test were employed to compare numerical variables between study groups. Category variables were determined using the chi-square test. *p* values were deemed statistically significant if they were less than 0.05.

**Result:** Between groups, the perioperative and demographic features were similar. The mean durations of sensory block (347.42 ± 91.06 versus 341.46 ± 68.84), motor block (308.36 ± 80.91 versus 310.84 ± 75.50), and overall analgesia (421.51 ± 121.62 versus 412.40 ± 107.0) minutes did not show a statistically significant difference between the groups. In addition, there was no significant difference (*p* > 0.05) in postoperative analgesic use, initial analgesia rescue time, or pain severity, as measured by the Numerical Rating Scale (NRS). The addition of dexamethasone did not result in any issues, nor was there a statistically significant difference in the onset time between the two groups.

**Conclusion:** Dexamethasone at a dose of 4 mg extends the duration of sensory, motor, and overall analgesia in a manner similar to that of 8 mg dexamethasone with comparable durations for both the initial analgesic request and overall analgesic use.

## 1. Introduction

One of the most well-known and often used anesthetic methods for orthopedic surgery is spinal anesthesia. The procedure involves injecting an anesthetic into the spinal cord, typically in the intrathecal area, to cause lower body anesthesia. It is an easy-to-use, reasonably priced treatment that has a high success rate for postoperative analgesia and complete sensory and motor block [1].

A number of spinal adjuvants have been used to extend postoperative analgesia and enhance the quality of spinal anesthesia. Adjuvants such as epinephrine, fentanyl, morphine, clonidine, ephedrine, pethidine, dexmedetomidine, ketamine, midazolam, neostigmine, and magnesium sulfate are, therefore, employed in addition to local anesthetics [2]. However, these can also result in negative side effects such as sleeplessness, nausea, vomiting, rash, respiratory depression, hypertension, hypotension, and psychotomimetic symptoms [3].

By lowering inflammation, preventing nociceptive C-fiber transmission, and inhibiting ectopic neuronal discharge, dexamethasone reduces pain. Although the exact mechanism underlying sustained analgesia following treatment with 4 mg is unknown, it is believed to be mediated by preventing the synthesis and release of inflammatory mediators [4] Therefore, medications with few adverse effects and extended analgesia are frequently sought. In addition, there must be a way to extend the analgesic benefits of spinal anesthesia without adverse effects.

In orthopedic surgery, the neuraxial administration of bupivacaine is expected to produce superior patient-oriented results, and it has been recommended as the preferred approach [5]. However, anesthesia professionals may argue against using this method due to the short duration of action of intrathecal local anesthetics [6]. Perioperative pain management remains a major concern both during and after orthopedic surgery. For half of the patients, standard pain management techniques do not provide sufficient analgesia, and postoperative discomfort might make it more difficult for patients to recover [6].

Most studies comparing different dosages of intrathecal dexamethasone have inconsistent results, despite the fact that it appears to be an efficient adjuvant to spinal bupivacaine because it prolongs the duration of analgesia, maintains a steady hemodynamic profile, and causes few adverse effects. Therefore, more research is needed to determine the ideal dosage and long-term safety of intrathecal dexamethasone [7]. Despite the long duration of intrathecal administration of dexamethasone, the combination of intrathecally administered bupivacaine has not been thoroughly evaluated. Furthermore, there is debate about whether administering dexamethasone at a dose of 8 mg offers any benefits over administering it at a dose of 4 mg. Doses variation is a key strategy currently being used to achieve postoperative pain control for patients receiving spinal anesthesia; however, more research is required. Our findings may serve as a baseline for future research, illustrate potential analgesic methods for postoperative pain management in orthopedic patients, and offer alternate approaches for improving the effectiveness and duration of spinal anesthesia.

## 2. Materials and Methods

### 2.1. Study Design, Period, and Area

Institutional-based prospective cohort study design was conducted from June 2021 to October 2021. The study was conducted at Sodo Christian Hospital (SCH) located in Wolaita Sodo, southern of Ethiopia. The hospital provides orthopedic, surgical, gynecologic, obstetric, and many other health services. The hospital has 140 beds and six operating rooms and performs 1572 elective orthopedic surgeries annually on average. The study was registered at https://www.researchregistry.com with the UIN: research registry 9396.

### 2.2. Inclusion Criteria

The study included elective orthopedic patients who were between the ages of 18 and 65, classified as Class II by the American Society of Anesthesiologists (ASA), and who had spinal anesthetic induction with 0.5% hyperbaric bupivacaine.

#### 2.2.1. The Exclusion Criteria

✓ Failed spinal anesthesia, spinal anesthesia other than bupivacaine with dexamethasone, drug addict, and patient receiving long-term steroid therapy were excluded from the study.

### 2.3. Sample Size Determination

Based on the mean score of sensory duration in minutes, two separate sample size formulas were applied to achieve an equal sample size, which produced the biggest sample size among the groups. The study was designed with a power of 80%, or 0.84, Type II error of Z*β* = 20%, and Type I error of Z*α*/2 = 5%, or 1.96. Since there has not been a published study on this subject in our nation before, the sample size was determined using the results of earlier studies. A study conducted in Iraq revealed that in the dexamethasone lower-dose group, the mean duration of sensory block time in minutes was 284.63 ± 64.85, whereas in the low-dose group, it was 313.37 ± 70.53 [8]. This sample size was calculated with G-power 3.1.9.2.

(1) $$\begin{array}{c} n=\frac{\left(S_{2}^{1}+S_{2}^{2}\right)\;{\left({Z\text{⁣}}_{1}+{Z\text{⁣}}_{2}\right)}^{2}\;}{{\left({\mu \text{⁣}}_{1}-{\mu \text{⁣}}_{2}\right)}^{2}}. \end{array}$$

For each group, *n* is the sample size; *z*_1_ is 1.96 for a 5% error (95% confidence level); *z*_2_ is 0.84 for 80% power; *S*_1_ represents the standard deviation of the low-dose dexamethasone group; *S*_2_ represents the middle-dose dexamethasone group; *μ*_1_ represents the mean of the low-dose dexamethasone group; and *μ*_2_ represents the mean of the middle-dose dexamethasone group.

(2) $$\begin{array}{c} n=\frac{\left({\left(64.85\right)}^{2}+{\left(70.530\right)}^{2}\right)\;{\left(1.96+0.84\right)}^{2}}{{\left(284.63-313.37\right)}^{2}}\\ =\frac{\left(4205.5225+4974.4809\right)\;\left(7.84\right)}{825.9876}\\ =\frac{71,971.226656}{825.9876}=89. \end{array}$$

A total of 178 adult patients participated in the study, with *n*_1_ = *n*_2_ = 89 participants in each group.

### 2.4. Sampling Technique

From situational analysis, SCH provides approximately 352 patients' study period. A systematic random sampling procedure was utilized to get the requisite sample size orthopedic services under spinal anesthesia annually. Consequently, the skip interval *K*=*N*/*n* = 352/178 = 2 was determined, and a lottery was used to select the first participant (random start). After that, from the daily surgery schedule list, every subsequent patient was included in this study.

### 2.5. Data Collection Tools and Procedure

Prior to collecting data, data collectors received training that comprised a quick explanation of the Numerical Rating Scale (NRS) scoring system and hands-on demonstration. Data were gathered using pretested questionnaires in 5% of the WSUCSH population. All patients who volunteered to participate in the trial and were scheduled for elective orthopedic surgery after giving their informed consent were thoroughly screened prior to surgery by getting a medical history and looking over their medical records. The patient was taught how to self-report pain using the eleven-point NRS, which ranges from 0 to 10, the morning before surgery. Following the patients' arrival in the operating room and the implementation of the standard hospital monitoring protocol, all patients were given spinal anesthesia between the L3-L4 and L4-L5 interspace using a midline approach. Four milliliters of 0.5% bupivacaine were combined with two different doses of dexamethasone, and either a 24- or 25-gauge spinal needle was used, depending on the preference of the attending anesthetist. Following this, all patients were placed in a supine posture, and their degree of feeling was evaluated using a pin prick test, which involved placing a needle with a short bevel along each side of the midaxillary line. The level of sensory block was measured every 30 s for 20 min, after which it was periodically assessed until the procedure was completed. Following that, the required intraoperative data were documented.

Patients were moved from the operating room to the surgical ward after the surgery. The relevant orthopedists or the resident frequently documented pain treatment as routine and PRN basis for ward nurses if a spinal anesthetic block was performed. The accountable ward nurses followed up on a regular basis. Every analgesic or other drug administered, together with its dosage and timing, will be recorded, along with the hemodynamic parameters (PR, BP, SpO_2_, and temperature). The SCH followed the WHO postoperative pain management guidelines, which call for treating light pain with NSAIDs (mostly + week opioids, tramadol) and severe pain with NSAIDs (diclofenac) + week opioids, tramadol + any powerful opioids that are available or an epidural catheter, mild pain (NSAIDs (diclofenac)/aspirin/PCM), Both groups' postoperative pain severity was evaluated using an NRS score. The horizontal lines that make up the scale go from 0 (no pain) to 10 (severe agony), or the worst suffering that can be imagined. On the NRS, patients were asked to rate their level of pain on a scale from 1 to 11. There was mild pain (NRS: 1–3), moderate pain (NRS: 4–6), severe pain (NRS: 7–10), and no discomfort (NRS: 0). The first NRS score for the procedure was obtained in the fourth hour and then in the sixth, eighth, twelve, and twenty-four hours. The pain score was computed following a deliberate cough or movement (dynamic NRS), as well as during a period of silent breathing or while at rest (static NRS). Both the total amount of analgesic medication used and the time it took for each patient to make their initial request were noted on the chart. During the pain assessment, the following parameters were measured: heart rate, mean arterial blood pressure, breathing rate, SpO_2_, and analgesic demand. Any postoperative side effects, such as nausea, vomiting, or shivering, were noted and brought to the attention of the doctors who were on call.

### 2.6. Data Processing and Analysis

**Figure 1:**
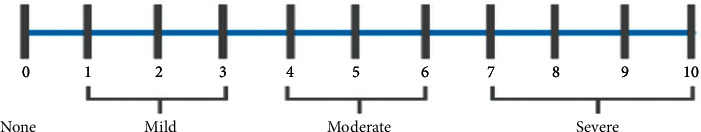
NRS score: NRS score adopted from the national initiative on pain control (NIPC).

After manually verifying that the data were accurate, it was coded, imported into EpiData Version 4.46, exported into SPSS Version 25, and examined again. The statistical analysis was performed with SPSS Version 25. The Shapiro–Wilk test was used to examine data distributions, and Levene's test for equality of variance was used to verify homogeneity of variance. The Mann–Whitney test and the independent sample *t*-test were employed to compare numerical variables between study groups. The chi-square test is applied to categorical or discrete variables. For regularly distributed data, the data are expressed as the mean ± SD and for skewed data, as the median ± inter quartile range. Categorical variables were described in terms of frequency and percentage. A 95% confidence interval and a *p* value of less than 0.05, along with 80% power, were deemed statistically significant (Figure 1).

## 3. Results

### 3.1. Participant Sociodemographic and Perioperative Characteristics

There were 178 patients in all, with 89 patients in each group and a 100% response rate based on whether they received 8 mg or 4 mg dexamethasone added to intrathecal bupivacaine for effectiveness of perioperative analgesia. The groups were found to have similar participant demographic and perioperative characteristics. The majority of patients were ASA I (85.4%) and males (77%). Lower leg surgery was the common type of the procedure (56.2%) in exposed and less-exposed groups. The results of the chi-square test indicated that, other from sex, which had a *p* < 0.05 difference, there was no statistically significant (*p* > 0.05) difference between the groups as shown in Table 1.

### 3.2. Preoperative Vital Signs

Preoperative vital signs expressed in the mean ± SD were comparable between the exposed and less-exposed groups. As the independent *t*-test results showed, there were no statistically significance (*p* > 0.05) difference between the groups (Table 2).

### 3.3. Characteristic of Spinal Anesthesia and Duration of Analgesia Between the Groups

As chi-square test result in Table 3 indicated that there was no significant difference between two groups regarding spinal blocks characteristics with exception of level of block with *p* = 0.003. The majority of block level was up to T-10 (83.2%) and the results between the groups were comparable (Table 4).

### 3.4. Characteristic of Spinal Anesthesia and Duration of Analgesia Between the Groups

An independent-samples *t*-test was conducted to compare duration of sensory, motor, and total analgesia time score between two groups and showed nonsignificant results with their respective *p* values with the value as shown in Table 3.

### 3.5. Comparison of Postoperative Pain Severity by Numeric Pain Rating Scale at Rest

**Figure 2:**
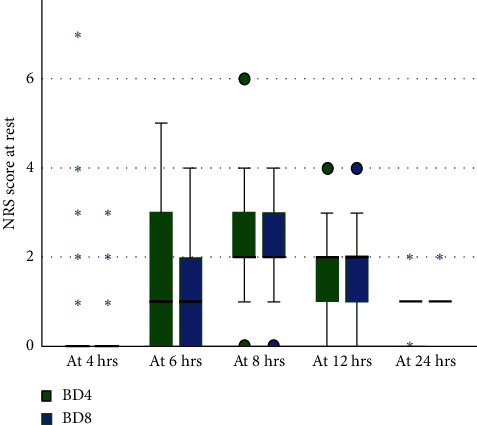
Comparison of postoperative pain severity by NRS score at rest between at Sodo Christian Hospital from June to October 2021, Wolaita Sodo, Ethiopia.

In order to ascertain if the NRS scores of the two groups—BD8 (*n* = 89) and BD4 (*n* = 89)—were different, a Mann–Whitney *U* Test was performed. The results showed that, for the 24 h that the groups were at rest, the difference in NRS ratings was not statistically significant (*p* > 0.05). NRS scores of BD8 and BD4 groups were comparable at all time during 24 h at rest as shown in Figure 2.

### 3.6. Comparison of Postoperative Pain Severity by Numeric Pain Rating Scale on Movement

**Figure 3:**
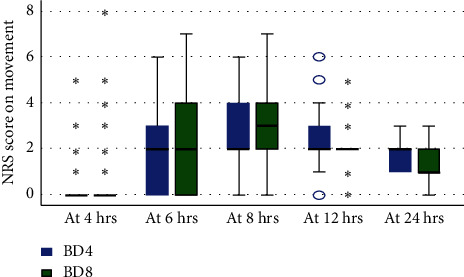
Comparison of postoperative pain severity by NRS score at movement b/n the groups at Sodo Christian Hospital from June to October 2021, Wolaita Sodo, Ethiopia.

A Mann–Whitney *U* Test was conducted to determine if there were differences in the NRS score between the groups: BD8 (*n* = 89) and BD4 (*n* = 89) and it revealed that the difference in NRS scores was not statistically significant during 24 h (*p* > 0.05) between the groups at voluntary movement. NRS scores of BD8 and BD4 groups were comparable at all time during 24 h on movement as shown in Figure 3.

### 3.7. Comparison of Time to First Analgesia Request Between Groups

**Figure 4:**
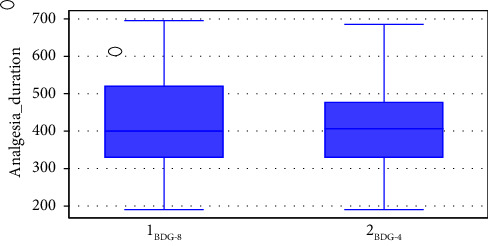
Time to first request analgesia in each group compared at Sodo Christian Hospital from June to October 2021, Wolaita Sodo, Ethiopia.

In terms of the time to first analgesia request, the independent sample *t*-test result indicates that the BD4 group prolongs the analgesia duration in a comparable way, with a mean and SD of 412.40 ± 107.01 minutes as opposed to 421.51 ± 121.62 minutes in the BD group (*p* = 0.597).  Figure 4.

### 3.8. Comparison of Total Analgesics Consumptions Between the Groups

The total number of analgesics consumed in a 24-h period did not yield a statistically significant result from the Mann–Whitney *U*-test (*p* > 0.05). It was discovered that the groupings were similar (Table 5).

### 3.9. Comparison of Each Group's Intraoperative Hemodynamic Response

Before and after spinal block, the hemodynamic response in each group was similar in terms of both the pulse rate and mean arterial pressure, with the exception of the pulse rate at 40 and 50 min (*p* = 0.037 and *p* = 0.027, respectively) (Table 6).

### 3.10. Comparison of Postoperative Hemodynamic Response Between the Groups

When the recovery room and ward were reached, the postoperative vital signs were collected right away. As can be seen, the data at 4, 6, 8, 12, and 24 h after surgery were not statistically significant (*p* > 0.05) (Table 7).

### 3.11. Comparison of Side Effects Between the Groups

No side effects were reported from intrathecal injection of dexamethasone and bupivacaine during the 24-h period after surgery, as no patient experienced hypotension, bradycardia, shivering, and PONV between the groups.

## 4. Discussion

All spinal blocks done in this study were effective, and confounding variables such as baseline, intraoperative, and postoperative vital signs, type of operation, duration of surgery, ASA status, and demographics (except gender) were similar in both groups. The two groups do not differ in terms of the spinal block duration, time to first request analgesia, pain intensity, or total analgesic use over a 24-h period.

According to our study, there was no significant difference between the 8 mg and 4 mg groups in terms of the mean time to length of sensory block and motor block (*p* > 0.05). The studies by Mohammad, Lotfy, and Mostafa [8], Bravo et al. [9], and Knezevic, Anantamongkol, and Candido [10] found, in line with our findings, that there was no statistically significant difference in the mean time to duration of sensory block and motor block between different doses of dexamethasone groups during caesarian section under spinal anesthesia and in the ultrasound-guided infraclavicular block (*p* > 0.05).

In lined with a study done in the USA, dexamethasone added to bupivacaine greatly extended the length of the motor block and enhanced the degree of analgesia after interscalene block, which is consistent with our findings. The duration of motor block and analgesia did not differ between low- and high-dose dexamethasone [11].

In contrast our finding, a study done by Panjai Inphum [12] reported that the duration of sensory loss in Dex4 group was longer than that of Dex2 group significantly (*p* < 0.05) but there was no difference in the duration of motor block. This discrepancy could be due to a difference in study design, variability in the population, and surgical procedures (their study included upper extremity blocks).

Another Indian study found that when added to 0.5% bupivacaine in supraclavicular brachial plexus block, dexamethasone at a dose of 8 mg considerably extends the duration of sensory and motor blockade and postoperative analgesia in comparison with dexamethasone at a dose of 4 mg. The start of sensory and motor inhibition occurs at the same time in all groups [13]. This disparity can result from different study designs, demographic heterogeneity, or surgical techniques (upper extremity blocks were included in their study).

The mean time for the first analgesic request between the two groups in our study did not differ substantially (*p* > 0.05). According to a research done by Albrecht, Kern, and Kirkham [14], Holland et al. [15], Abdel-Wahab [16], and Khan Alam Noor et al. [17], there was no difference in the mean time for the first analgesic request in low (4 mg) versus high (8 mg) dexamethasone as an adjuvant to bupivacaine in the regional block (*p* > 0.05).

A similar UK study on perineural dexamethasone for peripheral nerve blocks concluded that, with no significant side effects reported, a 4 mg dose of perineural dexamethasone prolongs the duration of analgesia following local anesthetic peripheral nerve blockade with efficacy comparable to an 8 mg dose. Although the exact mechanism underlying the sustained analgesia induced by 4 mg is unknown, it is thought to be mediated by blocking the manufacture and release of inflammatory mediators. Although dose-ranging studies are necessary, dexamethasone is an effective adjuvant to local anesthetics, which doctors should be aware of [14, 18].

Our results are supported by an Egyptian investigation that found no difference between the two doses of dexamethasone's first postoperative 24-h analgesic impact when added to a long-acting local anesthetic mixture in TAP blocks in patients following inguinal hernia surgery [16].

A Canadian study similarly demonstrated that intravenous dexamethasone at a dose of either 4 mg or 10 mg significantly prolongs, as compared with normal NS, the analgesic duration of a single-shot interscalene block following arthroscopic shoulder surgery. In addition, lowering the dose of dexamethasone from 10 mg to 4 mg decreased the incidence of perineal pruritus [19].

Our results are at odds with a research by Acharya [20] that compared the effects of 4 mg and 8 mg of dexamethasone with levobupivacaine in fascia iliac block and found that 8 mg was a better dose to utilize as an adjuvant with levobupivacaine in the FIB. As a more effective adjuvant to local anesthetics, 8 mg of dexamethasone can be suggested because of its ability to extend analgesia and effectively reduce the need for oral or intravenous analgesics. The variability of the medication type (levobupivacaine) and sample size differences employed in their investigation could be the cause of this discrepancy.

According to a UK study, intravenous dexamethasone, at a dose of 10 mg compared with 2.5 mg, had a higher effect in prolonging the duration of postoperative analgesia caused by interscalene brachial plexus blockage by at least 5 h. Shorter durations of analgesia may be achieved with lower intravenous dosages of dexamethasone [21]. The combination of GA and recorded outcomes for 48 h was employed in this investigation; dosage variability may be the cause of this discrepancy.

The median (IQR) of the two groups' 24-h total analgesic consumption did not show statistical significance (*p* > 0.05) with respect to total postoperative analgesic consumption. Studies by Turner et al. [22] showed that block duration, postoperative pain scores, and opioid consumptions in the 4 and 8 mg groups were equal, which is consistent with our findings.

Our research revealed that neither of the two groups receiving the varying doses of dexamethasone reported any incidence of side effects. This was similar to a prior Iranian study that examined the impact of two distinct hydrocortisone dosages on the degree of perioperative shivering during elective surgery performed under spinal anesthesia. That study found that the incidence of shivering was significantly lower in the hydrocortisone-treated group (1 mg/kg or 2 mg/kg) than in the control group. Patients on hydrocortisone, on the other hand, shivered less; yet, there was no discernible difference between the two treatment doses [23].

Contrary to what we found, a study on the effectiveness of intrathecal dexamethasone in preventing early complications from spinal anesthesia for elective caesarean sections found that adding 4 mg of intrathecal dexamethasone as an adjuvant for spinal anesthesia can significantly reduce shivering during the perioperative period and the frequency and manifestations of nausea and arterial hypotonia. The quality is not the same when 8 mg of intravenous dexamethasone is added [24]. The small sample size and varied research population employed in their investigation may be the cause of this discrepancy.

## 5. Limitation

As a cohort study, the confounders (observer bias) might not be controlled. In addition, we only spent a brief amount of time monitoring the patients—24 hours—during the postoperative period.

## 6. Conclusion

Dexamethasone in a dose of 4 mg extends the time of sensory, motor, and overall analgesia in a manner similar to that of 8 mg dexamethasone with comparable durations for both the initial analgesic request and overall analgesic use.

## Figures and Tables

**Table 1:** Demographic and perioperative characteristics at Sodo Christian Hospital from June to October 2021, Wolaita Sodo, Ethiopia.

Variables	BD8 group (*N* = 89)	BD4 group (*N* = 89)	*p* value
Age (years) M (IQR)	34 (23–48)	31 (23–43)	0.622
Sex (M/F *n*%)	67.5/32.5	86.5/13.5	0.002
Weight (kg) M (IQR)	65 (60–70)	61 (55–69)	0.600
Height (m) M (IQR)	1.72 (1.66–1.77)	1.70 (1.60–1.77)	0.328
BMI (kg/m^2^) M (IQR)	22 (20.2–23.7)	21.5 (20–23.8)	0.588
ASA (I/II *n*%)	83.1/13.9	87.6/12.4	0.396
PR ((mean ± SD)	84.45 ± 13.3	83.74 ± 12.55	0.715
MAP (mean ± SD)	90.28 ± 10.03	91.79 ± 7.92	0.268
Surgery duration (min) M (IQR)	110 (60–155)	90 (60–137.5)	0.119
Type of surgery			0.960
Upper leg surgery *n*(%)	32 (36)	31 (34.8)	
Lower leg surgery *n*(%)	50 (56.2)	50 (56.2)	
Ankle and foot surgery *n*(%)	7 (7.9)	8 (9.0)	

*Note:* Values are presented as *M* and IQR, the Mann–Whitney *U*-test was used. Values are presented as the frequency (%), the chi-square test was used. *p* < 0.05 is statistically significant.

Abbreviations: ASA, American Society of Anesthesiologist; BD8/4, bupivacaine with 8 mg/4 mg dexamethasone; M (IQR), median (interquartile range); M/F, male/female.

**Table 2:** Baseline vital signs' characteristics of the study participants between the groups at Sodo Christian Hospital from June to October 2021, G.C, Wolaita Sodo, Ethiopia.

Variables	BD8 group (mean ± SD)	BD4 group (mean ± SD)	*p* value
PR (bpm)	84.45 ± 13.3	83.74 ± 12.55	0.715
MAP (mmHg)	90.28 ± 10.03	91.79 ± 7.92	0.268

*Note:* Values are presented as the mean ± SD, the independent *t*-test was used. *p* < 0.05 is statistically significant.

Abbreviations: MAP, mean arterial pressure; PR, pulse rate.

**Table 3:** Characteristic of duration of spinal anesthesia and analgesia between groups at Sodo Christian Hospital from June to October 2021, Wolaita Sodo, Ethiopia.

Variables	BD8 group (mean ± SD)	BD4 group (mean ± SD)	*p* value
Duration of sensory block (min)	347.42 ± 91.06	341.46 ± 68.84	0.623
Duration of motor block (min)	308.36 ± 80.91	310.84 ± 75.50	0.833
Duration of total analgesia (min)	421.51 ± 121.62	412.40 ± 107.01	0.597

*Note:* Values are presented as the mean ± SD, the independent *t*-test was used. *p* < 0.05 is statistically significant.

**Table 4:** Comparison of the level of block, onset time, and intraoperative analgesia between groups at Sodo Christian Hospital from June to October 2021, Wolaita Sodo, Ethiopia.

Variables	BD8 group (*n* (%)	BD4 group (*n* (%)	*p* value
Level of block			0.03⁣^∗^
Up to T-10	82 (92.1)	66 (74.2)	
Up to T-8	4 (4.5)	6 (6.7)	
Up to T-6	3 (3.4)	17 (19.1)	
Onset of sensory block			0.248
<5 min	66 (74.2)	75 (84.3)	
5–10 min	21 (23.6)	13 (14.6)	
10–20 min	2 (2.2)	1 (1.1)	
Onset of motor block			0.359
<5 min	61 (68.5)	68 (76.4)	
5–10 min	23 (25.8)	19 (21.3)	
10–20 min 5(5.6) 2(2.2)			
Intraop analgesia			0.940
Yes	10 (21.2)	10 (21.2)	
No	79 (88.8)	79 (88.8)	

^∗^ *p* value < 0.05.

**Table 5:** Comparison of total analgesics consumptions at Sodo Christian Hospital from June to October 2021, Wolaita Sodo, Ethiopia.

Variables	BD8 group (M (IQR)	BD4 group (M (IQR)	*p* value
Opioid			
Tramadol	50 (50–125)	100 (50–125)	0.351
Nonopioid			
Aspirin	0 (0–600)	0 (0–300)	0.134
Paracetamol	2 (1–2)	2 (1–2)	0.286
Diclofenac	75 (75–75)	75 (75–75)	0.060

*Note:* Values are presented as the median and IQR (interquartile range). *p* < 0.05 is statistically significant.

**Table 6:** Comparison of intraoperative hemodynamic response between the groups at Sodo Christian Hospital from June to October 2021, Wolaita Sodo, Ethiopia.

Variables		BD8 group (mean ± SD)	BD4 group (mean ± SD)	*p* value
Before SA	PR (bpm)	86.62 ± 13.71	88.09 ± 13.61	0.473
	MAP (mmHg)	93.44 ± 13.65	95.28 ± 10.73	0.318

Immediately	PR (bpm)	85.93 ± 14.57	87.36 ± 14.32	0.511
	MAP (mmHg)	88.71 ± 14.21	91.19 ± 13.51	0.234

At 10 min	PR (bpm)	84.56 ± 14.70	86.07 ± 15.15	0.502
	MAP (mmHg)	85.83 ± 13.99	87.38 ± 14.31	0.466

At 20 min	PR (bpm)	83.36 ± 14.34	86.00 ± 13.85	0.213
	MAP (mmHg)	83.26 ± 12.59	86.71 ± 13.72	0.082

At 30 min	PR (bpm)	82.10 ± 13.08	84.70 ± 13.36	0.192
	MAP (mmHg)	83.11 ± 13.32	84.87 ± 13.07	0.377

At 40 min	PR (bpm)	81.29 ± 13.78	85.39 ± 12.23	0.037⁣^∗^
	MAP (mmHg)	83.99 ± 12.91	84.85 ± 13.59	0.664

At 50 min	PR (bpm)	80.79 ± 12.65	84.90 ± 11.93	0.027⁣^∗^
	MAP (mmHg)	84.65 ± 12.10	85.18 ± 12.13	0.772

At 60 min	PR (bpm)	82.28 ± 13.21	85.00 ± 12.84	0.165
	MAP (mmHg)	84.81 ± 11.26	86.28 ± 10.60	0.370

At 2 h	PR (bpm)	82.24 ± 13.51	84.20 ± 12.10	0.308
	MAP (mmHg)	86.73 ± 11.58	85.24 ± 10.66	0.372

At 3 h	PR (bpm)	82.17 ± 12.06	84.18 ± 11.86	0.264
	MAP (mmHg)	88.24 ± 10.24	86.12 ± 8.87	0.143

*Note:* Values are presented as the mean ± SD,

Abbreviations: MAP, mean arterial pressure; PR, pulse rate; SA, spinal anesthesia.

⁣^∗^*p* value <0.05 is statistically significant.

**Table 7:** Comparison of postoperative hemodynamic response between the groups at Sodo Christian Hospital from June to October 2021, Wolaita Sodo, Ethiopia.

Variables		BD8 group (mean ± SD)	BD4 group (mean ± SD)	*p* value
At 4 hrs	PR (bpm)	83.67 ± 11.65	84.11 ± 12.87	0.812
	MAP (mmHg)	86.03 ± 7.69	86.11 ± 8.81	0.949

At 6 hrs	PR (bpm)	88.01 ± 12.75	88.47 ± 12.92	0.811
	MAP (mmHg)	87.38 ± 10.04	88.37 ± 10.14	0.514

At 8 hrs	PR (bpm)	88.90 ± 12.59	89.31 ± 12.57	0.826
	MAP (mmHg)	89.09 ± 9.14	88.40 ± 10.69	0.646

At 12 hrs	PR (bpm)	85.58 ± 11.42	86.03 ± 12.22	0.804
	MAP (mmHg)	86.60 ± 7.71	88.63 ± 7.35	0.073

At 24 hrs	PR (bpm)	82.01 ± 12.35	81.57 ± 10.10	0.796
	MAP (mmHg)	85.60 ± 6.67	87.44 ± 6.67	0.067

*Note:* Values are presented as the mean ± SD. *p* value <0.05 is statistically significant.

Abbreviations: MAP, mean arterial pressure; PR, pulse rate.

## Data Availability

The datasets used/analyzed in the current study are available from the corresponding author upon reasonable request.

## Nomenclature

SCH: Sodo Christian Hospital
BMI: Body Mass Index
WSUCSH: Wolaita Sodo University Compressive Specialized Hospital
